# Supplementary material for: Climate change, livelihoods, gender and violence in Rukiga, Uganda: Intersections and pathways
Source: PLOS Glob Public Health. 2026 Jun 5;6(6):e0005393. doi: 10.1371/journal.pgph.0005393 (PMC13240901; doi:10.1371/journal.pgph.0005393)
Supplement: S1 Text — (DOCX) [file pgph.0005393.s001.docx]

**Supporting Integration of Livelihoods and Health Programmes**

**Topic Guide for key informant interviews/FGDs (English version)**

*Instructions to the interviewer are in italics*

***Introduction***

My name is/our names are _______________.

I am/we are a researcher working with a partnership of organisations: Rugarama hospital, the Uganda offices of the International Crane Foundation and a university in the UK called the London School of Hygiene & Tropical Medicine. I am/we are here to conduct a study that will ask questions about the sorts of challenges that you and others in your village have to deal with that affect your health, livelihood and local environment. And how you or others in your community have responded to these challenges.

I would like to begin by asking you to tell me a bit about yourself [*anything they want to say: age, education, children, occupation, things on their mind etc.]*.

*Then cover the following areas*

| **Topic** | **Example question (if you need to use it)/ instructions** | **Covered: Y / N** |
| --- | --- | --- |
| **Now I would like to ask you to describe to me what sorts of difficulties or challenges you and your community face at present?** | *Record each challenge in the order they are mentioned.*  *Probe:* health (e.g. covid, malnutrition, poor access to health services like ANC, FP, immunisations),  Livelihoods (changing farming because of land degradation, lower rainfalls, failing crops etc., poverty/hunger)  Environment (degrading wetlands, deforestation, changing climate/rainfall etc.)  [*get as much detail as you can on the nature of the different challenges]* |  |
| **Perceived connections between challenges** | Do you think there are connections between these different challenges? *Ask them to explain* |  |
| *Now take each mentioned challenge in turn and ask the following questions for each one.*  *NB: If people want to talk about Covid that is fine, let them talk about it (ask the questions below in relation to this) but then move on to the next challenge and say, OK now we are done with Covid so we should discuss another.* | | |
| **Thinking about the challenge of *[health/ livelihoods/environmental]* that you mentioned, what would you say has led to this challenge?** | *Probe: e.g.* any environmental changes, increase in numbers of people, anything else*?*  *Try to get a narrative explaining the triggers/links of things/events that led to the challenge, as they see it.* |  |
| **Are different people/groups in your community affected?** | Do these challenges affect everyone the same way or do they affect some people or groups in the community differently?  *Probe: Get them to explain how different people may be affected* |  |
| **How are people responding?** | How are you or people in your community responding to this *health/ livelihoods/environmental* challenge?  *Probe:* What actions have been/are being/will be taken? |  |
| **Are there differences in response between people or groups** | Do different people/groups respond differently or make decisions differently?  *Probe:* If there are differences, what do you think about this? |  |
| **Decision making** | Who makes decisions in this village about what to do about the *health/ livelihood/ environment* challenge you mentioned?  *Probe:* who is influential in this community? *(e.g. Church leaders, heads of families, local council leaders, group leaders)*  How are decisions made?  *Probe:* give examples, *e.g. through church or village meetings, within families, in village saving associations, LC1 level.* |  |
| **Is decision making changing?** | Thinking about the ways in which people respond to *health/ livelihood/ environment* challenges in this village, is anything done differently now from in the past?  *Probe further:* Why do you think things are done differently now? Is this helpful/not helpful? Do you think the way decisions are made will or should change in the future?  Do people move out of their communities more now or bring new influences from anywhere else? What effect does this have? |  |
| **Anything else to be done?** | Is there anything else that you think should be done, or that you think would help, to address this *health/livelihood/environment* challenge? [*Probe: for as much detail as you can]* |  |
| *Now return to the second challenge mentioned and repeat the above questions until all challenges have been covered.* | | |
| **Anything else or Questions** | Is there anything else you would like to mention? Or any question you would like to ask? |  |

Thank you very much for your time. I hope you have a good day.

[*give them a soda*]

Now make a short note about the interview/FGD

- the body language of the interviewee (did they seem comfortable or unhappy), were they happy talk, were there any interruptions etc.
- also about the local environment that it took place in: what was the house/hall and immediate environment like (e.g. animals/children around, weather, is the place clean/dirty, are there trees/planting around, anything else you think is noticeable)
