# Supplementary material for: Climate change, livelihoods, gender and violence in Rukiga, Uganda: Intersections and pathways
Source: PLOS Glob Public Health. 2026 Jun 5;6(6):e0005393. doi: 10.1371/journal.pgph.0005393 (PMC13240901; doi:10.1371/journal.pgph.0005393)
Supplement: Attachment — Submitted filename: Comments.docx [file pgph.0005393.s005.docx]

Respected Editor,

Thank you for the opportunity. Please find attached certain comments from my side to the authors -

1. Kindly mention the methodological orientation adopted for the study?
2. Discrepancy between number of participants in FGD mentioned in abstract and methods – (6 – 8 in abstract and 4 – 6 participants in methods)…Kindly make it uniform
3. Additional context on domestic violence and related statistics can be added in study setting
4. Details on steps taken to ensure internal validity/rigor to be mentioned – member checking, reflexivity
5. Give details of the parent project briefly
6. Any conceptual model/framework adopted to guide data generation/analysis?
7. What efforts were taken to address/refer victims of GBV once disclosed?
8. Socio - demographic details of the respondents could be added for better interpretation
9. Key themes are restated multiple times; Many dimensions of GBV (more details on each typology, coping strategies, prevention, etc) not elicited
