# Supplementary material for: Climate change, livelihoods, gender and violence in Rukiga, Uganda: Intersections and pathways
Source: PLOS Glob Public Health. 2026 Jun 5;6(6):e0005393. doi: 10.1371/journal.pgph.0005393 (PMC13240901; doi:10.1371/journal.pgph.0005393)
Supplement: Attachment — Submitted filename: Response to Reviewers Comments.docx [file pgph.0005393.s006.docx]

18 February 2026

Dear Editor,

**RE: RESPONSE TO EDITOR AND REVIEWERS’ COMMENTS**

We are pleased to resubmit our manuscript entitled *“Climate change, livelihoods, gender and violence in Rukiga, Uganda: intersections and pathways”* for further consideration in *PLOS Global Health*.

We sincerely thank the editors and reviewers for their careful evaluation and constructive feedback. We have revised the manuscript extensively in response to the comments provided and believe these revisions have substantially strengthened the clarity, rigour, and contribution of the work. Below, we provide a detailed, point-by-point response to all reviewer comments. For each comment, we indicate how and where the corresponding changes have been addressed in the revised manuscript.

We have also filled in the inclusivity in the global research questionnaire in the supporting documents for your reference.

1. Kindly mention the methodological orientation adopted for the study?

***Response: Thank you for this comment. We have stated that this was an exploratory, rapid qualitative research study in the methods section, both in the abstract and the main manuscript (under the Study Setting sub-section). We also added a Theoretical Orientation section at the end of the Introduction (see point 6 below). (Page 2, lines 33-34; page 8, line 163-164; page 7, lines 135-143)***

2. Discrepancy between number of participants in FGD mentioned in abstract and methods – (6 – 8 in abstract and 4 – 6 participants in methods)…Kindly make it uniform

***Response: Thank you for the careful observation. We confirm that the number of participants was between six and eight (6-8). This has been harmonised in the main manuscript. (Page 9, line 171)***

3. Additional context on domestic violence and related statistics can be added in study setting

***Response: The third paragraph of the Introduction details the situation in Uganda and further regional details have been added. Under Study Setting (in the Methods) the regional GBV prevalence has been added. There are no data for Rukiga itself. (Page 5, lines 80-86; page 8, lines 154-155***

4. Details on steps taken to ensure internal validity/rigor to be mentioned – member checking, reflexivity

***Response: Further details have been added in the Methods section in both Data Collection and Data Analysis sub-sections. (Page 9, lines 193-195). We added: The study strengthened internal validity and rigour by using standardised, pilot-tested FGD and KII guides as well as frequently debriefed among the team members to make sure that the data collected was coherent.***

5. Give details of the parent project briefly

***Response: This has been added to the Study Setting sub-section in the Methods. (Page 8, lines 159-164). We added text: The parent study involved formative research (reported in this paper) to identify the key health and livelihood challenges that people faced and inform intervention design. Cross-sector interventions (covering health, climate-smart agriculture and environmental protection) were then designed and evaluated (reported elsewhere) (38, 45) . This paper reports on the findings on GBV arising from the formative study, which was designed as a rapid, exploratory qualitative research study in four parish communities within Rukiga District (Nyakagabagaba, Kitojo, Kihanga-Sindi, and Burime).***

6. Any conceptual model/framework adopted to guide data generation/analysis?

***Response: Thank you for comment. We have added text in a new sub-section of the Introduction entitled “Theoretical Orientation” as follows; This study is informed by a gendered political ecology perspective, which conceptualises climate change not only as an environmental process but as one that is socially mediated through power relations, livelihoods, and gender norms (37). From this perspective, environmental stressors interact with existing gender inequalities and economic precarity to reshape household roles, authority, and conflict, with implications for gender-based violence (38). We also draw on intersectional and relational gender lenses to understand how these processes are experienced differently across social positions and how threatened masculine identities may mediate pathways to violence (39, 40). (Page 7, lines 135-143)***

7. What efforts were taken to address/refer victims of GBV once disclosed?

***Response:* *We have added a text under the Ethics sub-section in the Methods: The project also had an ethical obligation, once GBV was identified, to support those who reported it. Based on the findings reported in this paper, the MPT developed a GBV screening tool that was used at the clinic to assess disclosure by participants. Participants who disclosed any form of violence were referred to an NGO operating in the area that offers help and support to GBV survivors (including further counselling and other support services). (Page 12, lines 249-253)***

8. Socio - demographic details of the respondents could be added for better interpretation

***Response: We have included a new section on Demographic Characteristics at the beginning of the results section. (Page 12, lines 256-263):***

***Demographic characteristics***

***A total of 120 participants were included in the study, the majority of whom were female (64.2%). Nearly half were aged 18–35 years (47.5%), with the remainder distributed across 36–50 years (28.3%), 51–65 years (17.5%), and over 65 years (6.7%). Participants were predominantly Bakiga (96.7%). Most had primary (40.8%) or secondary education (40.0%), while 14.2% had tertiary education and 5.0% reported no formal education. Over half were married (55.8%), and farming or peasant work was the main occupation (61.7%). The majority identified as Protestant (55.8%) or Catholic (34.2%).***

9. Key themes are restated multiple times; Many dimensions of GBV (more details on each typology, coping strategies, prevention, etc) not elicited

***These have been rearranged and explained throughout the results section.***

Reviewer #1: Full Title:

Manuscript full title does not match with the short title. Full title reads "Climate change, livelihoods, gender and violence in Rukiga, Uganda: intersections and pathways". While short tile reads "Climate Change and Gender Based Violence". 'gender based violence' may not necessarily mean the same as 'gender and violence'. Authors should consider revising the wording in the full time if they meant gender based violence.

***Response: Thank you for the comment. We have renamed the manuscript, and it is now consistent.***

Abstract:

Inconsistency in FGD size, harmonize to consistent range across the manuscript. Author said "Between April and July 2021, we conducted 28 focus group discussions (FGDs), comprising 6-8 participants each (line 29-30" and in methods author said "From 20 April 2021- 02 July 2021 five focus group discussions (FGDs) were conducted in each community (28 in total) each consisting of four to six participants (lines 135-136)".
***Response: We have clarified that it was 6-8 participants per FGD. (Page 2, Line 34; page 9, line 171)***

clarify the GBV emergent theme. You said "This study, though not originally intended to focus on GBV, examines how it interconnects with poverty, shifting gender roles, alcoholism, environmental stress, and family planning dynamics." (lines 26-28). Consider adding a statement signalling GBV emerged inductively during data colletion and/or analysis.

***Response: We have added a statement to qualify it in the abstract (Page 2, lines 27-28; page 8, lines 163-164)***
Methods:
Revise the methods section to ensure the study can be reprodcible, and signal reliability of findings.

What study design did you use? not clear
***Response: We have clarified that it was an exploratory qualitative rapid assessment in the methods section. (Page 2, lines 33-34; page 8, line 164)***

Author said participants were " purposively selected... with the help of community leaders" (lines 140-141). Clearly elaborate the eligibility criteria and how the gatekeepers' influence was mitigated, and proper justification why 28 FGDs and 40 KIIs were sufficient. Talk about saturation, was maximum variation considered? and how?
***Response: We have added a text to explain how the community leaders were engaged explicitly. As follows:***

***“These community leaders were well known members of the community and had worked closely with the implementing partners. They were requested to identify equal numbers of men and women, from a range of backgrounds, ages and residences in the community. They were not present during the interviews, so that confidentiality was ensured.” (Page 9, lines 177-181)***

***We have already explained that the 28 FGDs represent maximum variation in the communities since the groups represented FGDs from each of 8 communities both sexes (male and female) and three age groups (18-25, 25-49, and 50+ years).***

***We have also added a text: FGD members were chosen from the community using community mobilisers to ensure nearly all villages were represented. In each community, we had two groups in each age category, except for the 50+ group, which had only one. For the KIIs, we conducted 10 per integrated site and were mobilised in the same way as the FGDs. Data saturation was achieved quite quickly, both within and between communities, but we aimed to conduct FGDs at every site, resulting in more than necessary, and the same applied to the KIIs. (Page 9, lines 184-190)***
Results:

Tag all quotes with data source (FGD or KII), sex, age to evidence diversity across the groups.

***Response: We confirm that all the quotes have their sources and age throughout the results section.***

Make sure all quotes are in clear quotations marks (lines 220-222). fix that for the entire results section and be consistent.

***Response: Thank you for the observation. We have harmonised it throughout the manuscript.***


Authors said "When describing their experiences and perceptions of poverty and its associated consequences including poor diets, sickness, and lack of ability to pay for healthcare and transport to medical facilities, most respondents explicitly identified poverty as a direct cause of GBV:" (lines 311-314). Revise the wording on participants' perceptions to avoid implying causality from qualitative data. Check the entire document for this including the abstract lines 36 to 41.
***Response: We have added the word “perceived” and have harmonised the entire manuscript.***
Ethics:
Include ethical committee name that gave ethical clearance for the study, also include the reference number and date.
***Response: Thank you for the observation. We have added a text with approving bodies: This study involved human participants and was approved by The London School of Hygiene and Tropical Medicine Research and Ethics Committee (24031), Makerere University School of Social Sciences Research and Ethics Committee (MAKSS REC 10.20.447/CR) and Uganda National Council for Science and Technology (HS1137ES). (Page 12, lines 242-245)***

describe safeguardings and referral procedures followed in the study if any.
***Response: Thank you for the comment we have added a paragraph at the end of the Ethics sub-section of the Methods to describe these***. ***(Page 12, lines 249-253): The project also had an ethical obligation, once GBV was identified, to support those who reported it. Based on the findings reported in this paper, the MPT developed a GBV screening tool that was used at the clinic to assess disclosure by participants. Participants who disclosed any form of violence were referred to an NGO operating in the area that offers help and support to GBV survivors (including further counselling and other support services).***

Conclusion:
The concept for this paper is timely and relevant. However several important elements require revision before the manuscript can meet PLOS Global Public Health Standards. Work on the clarity and consistency of the methods (study design was not clearly mentioned, there are several qualitative designs one can use, e.g. phenomenology, case study, etc. what design did you use?). PLOS Global Public Health guidelines on data sharing require that you provide some de-identified data, nevertheless authors stated that they would share data and the justification for that leaves much to be desired.

***Response: edits have been made in various parts of the Introduction and the Methods in response to this and other reviewer comments. Study Design is now clearly flagged in the first section of the Methods, and the theoretical orientation is explained at the end of the Introduction.***

***Regarding data sharing, our institution’s standard practice is that we do not make qualitative data publicly available, because it is never possible to fully anonymise it and retain its meaning and we must respect the approvals given to us by our participants (which did not include data use by third parties). We will consider bona fide research requests and make data available on a case by case basis.***

Reviewer #2: 1. Kindly mention the methodological orientation adopted for the study?

***Response: Thank you for the comment. We have added new sections in the Introduction (on theoretical orientation) and Study Design (in Methods). (Page 7, lines 135-143; page 8, lines 159-164)***

2. Discrepancy between number of participants in FGD mentioned in abstract and methods – (6 – 8 in abstract and 4 – 6 participants in methods)…Kindly make it uniform

***Response: Thank you. We have clarified this and added the information that it was between 6 and 8 participants. (Page 2, line 33-34; page 9, line 171)***

3. Additional context on domestic violence and related statistics can be added in study setting

Response: ***While no data on GBV are available for Rukiga itself, we have now added the regional prevalence (in Intro and Study Setting). (Page 5, lines 80-86; page 8, lines 154-158)***

4. Details on steps taken to ensure internal validity/rigor to be mentioned – member checking, reflexivity

Response:

***Response: details have been added in the Methods (see response to the same reviewer point above for further details). (Page 9, lines 193-195): The study strengthened internal validity and rigour by using standardised, pilot-tested FGD and KII guides as well as frequently debriefed among the team members to make sure that the data collected was coherent.***

5. Give details of the parent project briefly

***Response: This has been added to the Study Setting. (Page 8, lines 159-164): The parent study involved formative research (reported in this paper) to identify the key health and livelihood challenges that people faced and inform intervention design. Cross-sector interventions (covering health, climate-smart agriculture and environmental protection) were then designed and evaluated (reported elsewhere) (38, 45) .***

6. Any conceptual model/framework adopted to guide data generation/analysis?

***Response: We have added this in a new section in the Introduction: Theoretical Orientation. (Page 7, lines 135-143)***

7. What efforts were taken to address/refer victims of GBV once disclosed?

***Response: Text has been added to describe these procedures at the end of the Ethics sub-section in the Methods. (Page 12, lines 249-253): The project also had an ethical obligation, once GBV was identified, to support those who reported it. Based on the findings reported in this paper, the MPT developed a GBV screening tool that was used at the clinic to assess disclosure by participants. Participants who disclosed any form of violence were referred to an NGO operating in the area that offers help and support to GBV survivors (including further counselling and other support services).***

8. Socio - demographic details of the respondents could be added for better interpretation

***Response: Thank you. We have added a paragraph on the demographics in the manuscript as follows: A total of 120 participants were included in the study, the majority of whom were female (64.2%). Nearly half were aged 18–35 years (47.5%), with the remainder distributed across 36–50 years (28.3%), 51–65 years (17.5%), and over 65 years (6.7%). Participants were predominantly Mukiga (96.7%). Most had primary (40.8%) or secondary education (40.0%), while 14.2% had tertiary education and 5.0% reported no formal education. Over half were married (55.8%), and farming or peasant work was the main occupation (61.7%). The majority identified as Protestant (55.8%) or Catholic (34.2%). (Page 12, lines 256-263)***

9. Key themes are restated multiple times; Many dimensions of GBV (more details on each typology, coping strategies, prevention, etc) not elicited

***Response: We agree that our data do not capture all dimensions of GBV (e.g. a full typology, coping strategies, or prevention), because the parent study was not designed to focus on GBV and the interview guides did not include specific GBV questions or probes. We added a brief paragraph in the Results summarising the forms of GBV and limited references to coping/help-seeking that emerged spontaneously (Page 13, lines 279-282; page 13, lines 286-288; page 14, lines 298-300), and strengthened the Limitations (Page 30, lines 684-686) to clarify that, as GBV was not probed, we do not present a comprehensive typology, coping strategies, or prevention responses.***

Reviewer #3: Overall Comments
The paper takes a qualitative approach to “examine locally held perceptions of the relationships between climate and livelihood-related stressors and changing dynamcis, including the risk of Rukiga district. Climate change remains a global threat, with many countries and communities within Africa, ill prepared to adapt and mitigate the consequences. The paper is an attempt to paint a picture of climate-related impacts, particularly how gender-based violence, a persistent public health, socioeconomic and development issue is shaped by and influencing social, economic and environmental stressors.

In its current form, the paper need to be strengthened to get it to be sufficiently robust for publication in PLOS Global Public Health. The paper needs to be strengthened in at least three ways:

1) Overall, the paper needs to better contextualise their goal. Authors state in line 115 to 117, that their purpose is to understand locally held perceptions of the relationship between climate and livelihood-related stressors, and in several other sections, indicate make clear that, their original intention was not GBV, but undertook a thematic analysis on the latter. This can be confusing making it difficult for readers to follow. Authors need to clarify their focus – if it is on GBV, they may consider better contextualising their paper, especially in the introduction.
***Response: Thank you. We agree the framing was a bit unclear and we have now revised the Abstract and the aim statement at the end of the Introduction, and streamlined the wording in the Methods, to make clear that this paper presents a GBV-focused thematic analysis examining perceived relationships linking climate- and livelihood-related stressors, changing gender dynamics, and GBV risk in Rukiga District. (Page 2, lines 25-32; page 7, lines 127-134; page 8, lines 159-164; page 10, lines 209-213)***

As part of contextualising, authors may consider highlighting the initial primary research focus – this helps to provide context for readers to begin to appreciate how and why GBV took center-stage during the analysis. In doing so, it also provides an opportunity for authors to properly situate their contributions to the literature.
***Response: We agree and have added a brief paragraph in the Study Setting section of the Methods, and details in the Introduction, outlining the parent study’s initial focus and explaining how GBV emerged repeatedly in interviews/FGDs, which is why it became central to the analysis; we also use the Introduction to better situate the paper’s contribution to the climate–livelihood–GBV literature. (Page 2, lines 26-29; page 7, lines 127-134; page 8, lines 159-164)***

Other minor issues include:
• Authors make claims about projected exponential increase (line 51-52) and yet, do not support with any data. Similarly, authors may want to consider revising the sentence, as it appears redundant.
***Response***: ***We have deleted the word “exponential” and have revised the sentence. (Page 4, lines 57-58)***

• In line 55-57, it argued “Uganda’s vulnerability to climate change and climate-sensitive disasters is extremely high – it is not immediately clear to readers what this means. By which benchmark or metric are authors assessing Uganda’s vulnerability. Authors may consider revising to ensure clarity (also see lines 108-110 for punctuation issues).
***Response***: ***We have deleted this sentence and checked punctuation etc. throughout (Page 4, lines 61-64)***

• Lastly, the study takes place in Rukiga District – it would be helpful if authors provided some additional background context. Will the results be different, if the study was conducted in a different district rather than Rukiga? Basically, some discussions of the rationale and/or choice of the selected district is be useful.
***Response: Details have been added to the Study Setting section of the Methods (Page 8, lines 154-166)***

2) Overall, authors need to improve their methods by revising and clarifying, some of the sections. For example, under study setting (line 128-130), it is not clear if the concluding sentence is provided additional context for the prior statement. Authors may want to revise for clarity purpose.
***Response: The Study Setting section has been rewritten and further clarifying details added throughout the methods. (Page 8, lines 154-166)***

I. Reconcile the number of participants for FGDs – in the abstract, authors indicate 6-8 people form a FDG and in line 136, it says “…each consisting of four to six participants,…”.

***Response: This has been harmonised. (Page 2, line 34; page 9, line 171) and confirmed that participants were between 6 and 8.***

II. For both FGD and KII, it is useful to indicate and/or describe the demographic/characteristics of the people participating in the study (Perhaps, authors could outline their demographics by sex and age, and any other stratifier in the results section in a tabular format. How were participants selected, especially among the FGD participants?

***Response: This has been added in the methods section: (Page 9, lines 177-190). Demographics have been added at the beginning of the Results section (Page 12, Line 256-263).***

III. On ethics statement, although the data emanates from key informants and community members, authors do not indicate whether they sought ethnical approval for their study. If ethics was obtained, it is useful to indicate so.

***Response: We have now included the approving bodies in the manuscript. This study involved human participants and was approved by The London School of Hygiene and Tropical Medicine Research and Ethics Committee (24031), Makerere University School of Social Sciences Research and Ethics Committee (MAKSS REC 10.20.447/CR) and Uganda National Council for Science and Technology (HS1137ES). (Page 11-12, lines 242-245)***

IV. Regarding data collection, lines 172 to 173, authors indicate that “discrepancies in the coding were re-examined…”. It useful to explain how the independent assessor resolved discrepancies and reached consensus.

***Response: Further details on the coding and analysis process have been added. (Page 11, lines 222-238)***

V. In the data collection section (line 155 to 157), authors indicate that they “undertook a specific analysis of what participants said about GBV”. However, in the results, it is often not clear, the specific thematic issues or results arising from this analysis. Related to this and linked to the analysis, it is not clear to readers how the two main clusters (line 188 to 191) link to GBV. While lines 193 to 212, describe nature of GBV, for the most parts (for example, line 213 to 308), it is difficult to follow how GBV is an interconnector in the results being discussed. At times, it difficult to see, where the analysis departs from its original intended goal. Were the issues around climate change and environment among others emergent from the data?

***Response: Thank you, we agree this was not sufficiently clear. We clarified in the Methods (data collection section) that the GBV-focused analysis examined all GBV-related excerpts and the factors participants linked to GBV, with an emphasis on perceived pathways. In the Results, we added short linking sentences at the start (and brief summary lines at the end) of each of the two sections to make explicit how each cluster connects to GBV risk.(Page 10, lines 209-213; page 14, lines 298-300; page 14-15, lines 310-313; page 15, lines 326-328; page 15, lines 334-336; page 19, lines 428-429; page 20, lines 436-439; page 25, lines 559-561)***

3) Overall, the results section outlines some very interesting insights. However, I do feel this section can be deepened. In many instances, the narratives are often not immediately supported by the relevant quotes, linking to GBV.
• In line 230 – 323, authors reflect that the disruption to livelihoods leading to family instabilities and conflict, demonstrate how GBV is triggered. This assumption is challenging to sustain, considering that “unrest in families” and not having “peace in a home” do not necessarily connote GBV. Similar reflections are presented at line 306 (“...they both resort to quarrels…”), lines 316 to 320 (…start quarrelling and fighting…”) and (“…you fight with the woman”).

• Although authors indicate these are “euphemisms for GBV” (line 208) that participants use – without critical analysis, we risk painting a picture that may not be correct. For example, will readers be correct to assume, that in Ugandan context, such referencs always mean GBV?. To avoid readers assuming without appropriate understanding of context, authors may consider, making explicit any additional nunaces related to the quotations or contexts for this pharses, to clarify and make the links to GBV much clearer.
***Response: To respond to both points above, we revised the Results to avoid equating general household ‘conflict’ (e.g., ‘no peace’, ‘quarrels’) with GBV unless participants explicitly described violence or clearly framed the experience as GBV.*** ***(Page 14, lines 298-300)***

***We added a clarifying sentence in the Results to explain how we interpret euphemistic terms, and we adjusted the wording in the climate/livelihood section to describe increased stress and conflict, noting violence only where it is explicitly stated. We also added/relocated quotes that directly reference violence (e.g., “beating”) alongside the relevant interpretations to strengthen the evidentiary link in the results section as noted in the previous comment..***

Minor
• Line 199 – please clarify how and why unintended pregnancies is considered a form of GBV

***Response***: ***We have rewritten this – (page 13, line 279-282)***

• Line 208 to 209 – revise sentence – it is not clear what authors mean by throughout their experiences and perceptions

***Response: We revised line 199 to clarify that it was discussed in relation to GBV-related dynamics, specifically coercion and conflict/violence around family planning and reproductive decision-making. (Page 13, lines 286-288)***

• Line 211 – “GBV was raised during the discussions of a wide range of factors” – perhaps, useful to outline the contexts which GBV was raised

***Response:*** ***This has been added (Page 14, lines 310-314)***

Thank you so much for taking the time to review our manuscript and we hope that we have satisfactorily addressed the editorial and reviewers’ comments.

Kind regards,

Richard Muhumuza

Corresponding author.

[Richard.Muhumuza@mrcuganda.org](mailto:Richard.Muhumuza@mrcuganda.org)
