# Supplementary material for: Climate change, livelihoods, gender and violence in Rukiga, Uganda: Intersections and pathways
Source: PLOS Glob Public Health. 2026 Jun 5;6(6):e0005393. doi: 10.1371/journal.pgph.0005393 (PMC13240901; doi:10.1371/journal.pgph.0005393)
Supplement: Attachment — Submitted filename: Response to Reviewers Comments-R2.docx [file pgph.0005393.s007.docx]

19 March 2026

Dear Editor,

**RE: RESPONSE TO EDITOR AND REVIEWERS’ COMMENTS**

We are pleased to submit our second revision of the manuscript entitled *“Climate change, livelihoods, gender and violence in Rukiga, Uganda: intersections and pathways”* for consideration in *PLOS Global Health*.

We sincerely thank the editors and reviewers for their careful evaluation and constructive feedback.

We have revised the manuscript extensively in response to the comments provided and believe these revisions have substantially strengthened the clarity, rigour, and contribution of the work.

Below, we provide a detailed, point-by-point response to all reviewer comments.

We have also included Topic guides, a coding framework, and a manual workbook, which are available in the supporting documents for your reference.

Required revisions

1) Rigor and reflexivity

In your response to the reviewers, you emphasize pilot testing and team debriefing as strategies to enhance rigor. These are helpful, but they do not fully address the reviewer’s specific request regarding member checking and reflexivity/positionality.

Please revise the Methods (or a short reflexivity subsection) to include:

* Member checking: State explicitly whether participant/member checking was conducted.
* If yes, describe what was done and at what stage (e.g., returning summaries, validating themes, community feedback session).
* If no, state this transparently and briefly justify why (e.g., feasibility, safety/ethics, rapid design), and strengthen alternative credibility strategies (e.g., audit trail, reflexive memoing, peer debriefing, negative/discordant case consideration, triangulation logic where relevant).
* Reflexivity/positionality: Add a short paragraph describing:
* who collected the data (roles, relevant identities if appropriate),
* relationship to participants/communities (including any implementing partners),
* steps taken to mitigate power dynamics and interpretive bias, and
* how reflexive practices were incorporated (e.g., memoing, team discussions, analytic decisions).

**Response:** Thank you for identifying this. We have added a section: Positionality and Reflexivity statement on Page 11 Line 222-236

***Positionality and Reflexivity Statement***

*This study was conducted by a multidisciplinary team of experienced Ugandan and British researchers. The Ugandan team, who collected the data (RM, GN and JK), brought insider knowledge of the local culture, language, and community dynamics, which facilitated rapport-building, trust, and access to community members in the study sites. With no existing relationship to the community, the researchers acknowledged their outsider stance and engaged with openness. The British team members (MC, PZ, IC and SM), who were not involved in data collection, provided outsider perspectives that enhanced reflexivity during analysis and helped identify assumptions and biases that may have been embedded in interpretation. Reflexive practices, including weekly peer debriefings and member checking, were employed to critically examine how researchers’ positions, prior experiences, and beliefs influenced questioning strategies, probing, and interpretation of participants’ narratives. This combination of insider and outsider perspectives strengthened the credibility of the findings and contributed to a nuanced understanding of the challenges affecting people in these communities.*

2) Title consistency

There appears to be a remaining mismatch between the full title and the short title, and/or between the manuscript file and the submission system metadata (e.g., “gender and violence” vs “gender-based violence”). Please ensure:

* the full title and short title are aligned, and
* the title in the submission system exactly matches the title shown in the manuscript file.

**Response:** *Thank you, and this has been harmonised.*

3) Data Availability statement

We acknowledge the rationale for not sharing qualitative transcripts publicly, given their sensitivity and potential impact on participant safety. However, the Data Availability statement should be strengthened to maximize transparency and align with the journal's expectations.

Please revise the statement to specify clearly:

* what can be shared (e.g., de-identified excerpt set, codebook, analytic framework, thematic matrix, or other non-identifiable materials), even if full transcripts cannot be shared;
* how requests are handled (who adjudicates requests—named office/role/institution rather than an individual where possible);
* criteria for access (e.g., ethics approval, data use agreement, purpose/risks); and
* process/timeline for responding to requests.

If you cannot share any excerpted qualitative data at all, please state this explicitly and provide the strongest feasible alternative (e.g., codebook and analytic outputs).

**Response:** *We have attached the interview guides and coding framework to reflect what was asked in the field. We also attach our codebook showing the themes identified. Full original transcripts cannot be made available for further research because participants did not grant approval for this. Anonymised, excerpted data will be held in LSHTM’s data repository* [*LSHTM Data Compass*](https://datacompass.lshtm.ac.uk/) *and can be requested through this site by legitimate researchers. Requests will be considered on a case by case basis with criteria for access including possession of ethics approval, data use agreement, purpose etc. Response times are usually 2-3 weeks.*

4) Consistency checks

Please do a final consistency check for the following:

* FGD size: Ensure the stated group size is consistent throughout the manuscript (abstract, methods, results, tables/appendices if any).
* Sampling/geography wording: Ensure terms like “communities/parishes” and the number of sites are consistent throughout.
* Referral pathway wording: Ensure the safeguarding/referral description is consistent, clear, and accurately reflects what was implemented in practice (and does not imply default referrals that were not part of protocol).

Response: *Language has been checked and harmonised throughout.*

5) Clean tracked-changes + clean manuscript

To facilitate production, please upload:

* a clean, revised manuscript (no tracked changes), and
* a tracked-changes version that is carefully checked for accidentally duplicated paragraphs or repeated blocks inserted during revision.
Response: *We have checked all the references and harmonised the reference section of the manuscript.*

Optional but recommended refinements

* Design labeling clarity: Consider refining the study design description (e.g., “rapid qualitative inquiry” or “rapid assessment with thematic analysis”) and briefly clarifying the operational meaning of “rapid” (e.g., timeline or analytic approach) to reduce ambiguity for qualitative readers.
* Causal language sweep: Continue to avoid causal claims and ensure that the Results and Discussion sections consistently reflect perceived pathways (for example, “participants described…” or “participants perceived…”).

**Response:** *Thank you for this observation. We have harmonised these throughout the manuscript.*

Thank you so much for taking the time to review our manuscript and we hope that we have satisfactorily addressed the editorial and reviewers’ comments.

Kind regards,

Richard Muhumuza

Corresponding author.

[Richard.Muhumuza@mrcuganda.org](mailto:Richard.Muhumuza@mrcuganda.org)
