# Supplementary material for: Climate change, livelihoods, gender and violence in Rukiga, Uganda: Intersections and pathways
Source: PLOS Glob Public Health. 2026 Jun 5;6(6):e0005393. doi: 10.1371/journal.pgph.0005393 (PMC13240901; doi:10.1371/journal.pgph.0005393)
Supplement: Attachment — Submitted filename: Response to Reviewers Comments-R3.docx [file pgph.0005393.s008.docx]

06 April 2026

Dear Editor,

**RE: RESPONSE TO EDITOR AND REVIEWERS’ COMMENTS**

We are pleased to submit our second revision of the manuscript entitled *“Climate change, livelihoods, gender and violence in Rukiga, Uganda: intersections and pathways”* for consideration in *PLOS Global Health*.

We are sincerely grateful to the editors and reviewers for their consistent evaluation and constructive feedback.

We have revised the manuscript extensively in response to the comments provided and believe these revisions have substantially strengthened the clarity, rigour, and contribution of the work.

**Journal Requirements:**

If the reviewer comments include a recommendation to cite specific previously published works, please review and evaluate these publications to determine whether they are relevant and should be cited. There is no requirement to cite these works unless the editor has indicated otherwise. 

Please review your reference list to ensure that it is complete and correct. If you have cited papers that have been retracted, please include the rationale for doing so in the manuscript text, or remove these references and replace them with relevant current references. Any changes to the reference list should be mentioned in the rebuttal letter that accompanies your revised manuscript. If you need to cite a retracted article, indicate the article’s retracted status in the References list and also include a citation and full reference for the retraction notice.

***Resp:*** *We have reviewed the reference and hope all has been rectified.*

**Additional Editor Comments (if provided):**

The manuscript has improved considerably over the course of revision. The revised version demonstrates meaningful attention to the reviewers’ and editor’s comments, particularly in strengthening the framing, clarifying the methodological approach, expanding reflexivity, improving the discussion of safeguarding and referral processes, and providing a more nuanced interpretation of the relationship between livelihood stress, household conflict, and gender-based violence.
However, a small number of issues remain outstanding and should be addressed before the manuscript can be accepted.

**Response: Thank you**

First, the issue of member checking has still not been fully resolved. In the response letter, the authors indicate that “weekly peer debriefings and member checking” were undertaken. However, the manuscript does not clearly explain what the member checking entailed, whether it involved participants directly, and at what stage it occurred. Please revise the manuscript to state explicitly whether member checking was conducted and, if so, briefly describe the process. If the study did not include participant/member checking in the conventional sense, please remove that terminology and instead describe the specific credibility procedures that were used.

***Response:*** *Thank you for the comment. We have revised the reflective statement as follows: Reflexive practices, including weekly peer debriefings and internal checking of interpretations between researchers and programme staff, were used to examine how researchers’ positionalities and prior experiences shaped data collection and analysis. The study did not conduct participant member checking in the conventional sense. Instead, credibility was supported through these internal reflexive processes and through dissemination meetings with community members and district stakeholders. It is during these meetings that preliminary interpretations were shared, and feedback was gathered prior to finalising the results. This combination of insider and outsider perspectives strengthened the credibility and depth of the analysis. Page 12 Line 250-258 Tracked manuscript.*

Second, the Data Availability statement remains inconsistent across the submission materials. The response letter suggests a clearer controlled-access pathway for anonymized excerpted data, including institutional oversight and access procedures. However, the manuscript/front matter still appears to include older wording indicating that data are not publicly shared and referring only to the interview guide and coding framework in the supporting materials. Please ensure that the Data Availability statement is fully harmonized across the manuscript and submission metadata and clearly specifies what data are available, through what mechanism, under what conditions, and with what process for review and release.

**Response:** *We have attached the interview guides to reflect what was asked in the field. We also attach our codebook showing the themes identified. Full original transcripts cannot be made available for further research because participants did not grant approval for this. Anonymised, excerpted data will be held in LSHTM’s data repository* https://datacompass.lshtm.ac.uk/ *and can be requested through this site by legitimate researchers. Requests will be considered on a case-by-case basis with criteria for access including possession of ethics approval, data use agreement, purpose etc. Response times are usually 2-3 weeks. All data requests for select excerpts to clarify any ambiguities should be sent to the senior author Susannah Mayhew (Susannah.Mayhew@lshtm.ac.uk)*

Finally, please conduct one last careful consistency check of the clean manuscript before resubmission. The compiled editorial PDF suggests some remaining inconsistencies across versions, and all text, metadata, and supporting statements should now be fully aligned.

**Response:** Thank you for the careful observation. We have carefully reviewed the manuscript and aligned everything.

We hope that our revision meets the expectations of the journal and will now be accepted for publication.

Kind regards,

Richard Muhumuza

Corresponding author.

Richard.Muhumuza@mrcuganda.org
