# Supplementary material for: Climate change, livelihoods, gender and violence in Rukiga, Uganda: Intersections and pathways
Source: PLOS Glob Public Health. 2026 Jun 5;6(6):e0005393. doi: 10.1371/journal.pgph.0005393 (PMC13240901; doi:10.1371/journal.pgph.0005393)
Supplement: Attachment — Submitted filename: Response to Reviewers Comments-R4.docx [file pgph.0005393.s009.docx]

29 April 2026

Dear Editor,

**RE: RESPONSE TO EDITOR AND REVIEWERS’ COMMENTS**

We are pleased to submit our fourth revision of the manuscript entitled *“Climate change, livelihoods, gender and violence in Rukiga, Uganda: intersections and pathways”* for consideration in *PLOS Global Health*.

We are sincerely grateful to the editors and reviewers for their consistent evaluation and constructive feedback.

We have revised the manuscript extensively in response to the comments provided below and believe these revisions have addressed all the concerns raised.

**Journal Requirements:**

If the reviewer comments include a recommendation to cite specific previously published works, please review and evaluate these publications to determine whether they are relevant and should be cited. There is no requirement to cite these works unless the editor has indicated otherwise.

***Response:*** *Thank you for the careful review. I have realised that one of the references in the paper was a preprint and this has been removed from the manuscript. The reference removed was initially 27 (Goudet J‑M, Binte Arif F, Owais H, Uddin Ahmed H, Ridde V. Intersecting Vulnerabilities: Unravelling the Complex Relationship between Women, Climate Change, and Mental Health in Bangladeshi Communities. medRxiv preprint, 2023.) Page 5 Line 87*

Please review your reference list to ensure that it is complete and correct. If you have cited papers that have been retracted, please include the rationale for doing so in the manuscript text, or remove these references and replace them with relevant current references. Any changes to the reference list should be mentioned in the rebuttal letter that accompanies your revised manuscript. If you need to cite a retracted article, indicate the article’s retracted status in the References list and also include a citation and full reference for the retraction notice.

***Response:*** *We have checked the references after removing the preprint above and feel that they are now complete and correct.*

**Additional Editor Comments (if provided):**

There is still a potentially important sample-size inconsistency. The manuscript says there were 28 FGDs with six to eight participants each, plus 40 KIIs. That would imply at least 168 FGD participants plus 40 KII participants. But the Results section says: “A total of 120 participants were included in the study.”

***Response:*** *Thank you for highlighting this inconsistency. We apologise for the lack of clarity. While 28 FGDs (6–8 participants each) and 40 KIIs were conducted, complete demographic data were only available for 120 participants, as these were not consistently recorded across all FGDs. The figure of 120, therefore, reflects only participants with complete demographic information, not the total number who took part in the study. We have revised the demographics as follows; Page 12 Line 260-2264*

*A total of 28 focus group discussions (FGDs), each with approximately 6–8 participants, and 40 key informant interviews (KIIs) were conducted. Demographic information, however, was not consistently collected for all FGD participants. The characteristics presented here therefore reflect the 120 participants for whom complete demographic data were available. Among these, the majority were female (64.2%). Nearly half were aged 18–35 years (47.5%), followed by 36–50 years (28.3%), 51–65 years (17.5%), and over 65 years (6.7%). Participants were predominantly Bukiga (96.7%). Most had primary (40.8%) or secondary education (40.0%), while 14.2% had tertiary education and 5.0% had no formal education. Over half were married (55.8%), farming or peasant work was the main occupation (61.7%), and most identified as Protestant (55.8%) or Catholic (34.2%).*

We hope that our revision meets the expectations of the journal and will now be accepted for publication.

Kind regards,

Richard Muhumuza

Corresponding author.

Richard.Muhumuza@mrcuganda.org
